# OPREVENT (Obesity Prevention and Evaluation of InterVention Effectiveness in NaTive North Americans): Design of a Multilevel, Multicomponent Obesity Intervention for Native American Adults and Households

**DOI:** 10.1093/cdn/nzz009

**Published:** 2019-02-13

**Authors:** Leslie C Redmond, Brittany Jock, Preety Gadhoke, Dorothy T Chiu, Karina Christiansen, Marla Pardilla, Jacqueline Swartz, Harrison Platero, Laura E Caulfield, Joel Gittelsohn

**Affiliations:** 1 Johns Hopkins University Bloomberg School of Public Health, Baltimore, MD; 2 University of Alaska Anchorage, School of Allied Health, Dietetics and Nutrition Department, Anchorage, AK; 3 St. John's University, Department of Pharmacy Administration & Public Health, Fresh Meadows, NY

**Keywords:** Native North American, prevention, obesity, chronic disease, multilevel intervention

## Abstract

Obesity prevalence is high in Native American (NA) adults, and there is a critical need to establish and implement evidence-based social, behavioral, and policy interventions that are theoretically informed. The use of multilevel, multicomponent (MLMC) interventions has been shown to be an effective strategy for comprehensive health behavior change; however, there is little guidance available in the literature to facilitate implementation in this underserved and understudied population. To decrease obesity and related comorbidities in NA adults, an MLMC intervention called OPREVENT (Obesity Prevention and Evaluation of InterVention Effectiveness in NaTive North Americans) was implemented in 5 rural NA communities to modify the food-purchasing environment, improve diet, and increase physical activity (PA). Five NA communities across the Upper Midwest and Southwest United States were randomly assigned to Immediate (*n* = 3) or Delayed (*n* = 2) Intervention. OPREVENT was implemented in Immediate Intervention community food stores, worksites, schools, and media over 1 y. A community-randomized controlled trial was used to evaluate intervention impact in adults at the individual and institutional levels, with individual-level data being collected on diet, PA, and psychosocial variables at baseline and follow-up; and institutional-level data being collected on food stores, worksites, and schools, media, and process measures. The OPREVENT intervention was one of the first MLMC obesity interventions in this population and provides evidence-based practices for future program development. The purpose of this article is to describe the design, implementation, and evaluation of OPREVENT.

This trial was registered at isrctn.com as ISRCTN76144389.

## Introduction

Native Americans (NAs) and Alaska Natives (ANs) experience disproportionately high prevalence of obesity (1). Recent decades have seen a rapid nutrition transition in these populations, from nutrient-dense subsistence foods to energy-dense prepared and packaged foods often high in fat and refined carbohydrates that are associated with increased prevalence of obesity and other chronic diseases (2). A physical activity (PA) transition has also been noted, as traditional forms of PA associated with subsistence lifestyles are becoming less frequent and hunting and gathering are no longer necessary for survival, towards greater physical inactivity and lower energy expenditure (3, 4).

The historical context of colonization and land dispossession has shaped the food landscape and the built environment of NA reservation communities (5–7). Healthy food access is low, and the USDA Food Desert Locator tool places nearly all NA reservations in food deserts (8). Food insecurity is high, ranging from estimates of 16.3% of NA households without children, to 76.7% of households in the Navajo Nation (9, 10). NA populations suffer from the highest prevalence of poverty of any race or ethnicity in the United States (11). At the start of the current decade, it was estimated that 27% of all NAs lived below the poverty line (11). High poverty and food insecurity are related to poor-quality diet and low PA, in addition to an increased burden of both physical and psychological diseases (12–16). Poverty and food insecurity can also affect the food environment, which is a particularly important factor affecting food access and influencing dietary choices and food-purchasing behaviors in NA communities. Many NA communities only have small food stores in the community; these small stores have a limited selection of healthy food options and poor-quality fruits and vegetables (17, 18). The often rural and geographically isolated locations of NA communities lead food stores within these communities to stock nonperishable often highly processed, high-fat, and high-energy foods (19). In addition, the Food Distribution Program on Indian Reservations (FDPIR), an income-based federal supplementary food assistance program upon which many NA individuals and families depend (20), has been found to offer food packages that fall short of national guidelines for dietary quality. A recent study calculated the Healthy Eating Index-2010 scores for FDPIR food packages, and although the FDPIR food packages were found to score higher than other federal food assistance and nutrition programs, the total score was still quite low (66 out of 100) (21).

There are several lifestyle risk factors also contributing to this increased prevalence of overweight, obesity, and chronic disease in NAs and ANs, including excess energy intake (22, 23), high fat intake (22, 23), and low PA (1, 24). The Strong Heart Dietary Study Phase II, conducted to investigate the intake of dietary nutrients that contribute to cardiovascular disease in NA and AN populations, reported that NA adults aged ≥45 y from Arizona, North and South Dakota, and Oklahoma exceeded Dietary Reference Intakes and American Heart Association guidelines for carbohydrates, protein, and sodium (25, 26). Researchers also found that NA women consumed lower amounts of folate and vitamins A and C, and NA men consumed lower amounts of vitamins A, B-6, and E (25, 26). Less than half of participants met the USDA Healthy People 2000 guidelines for reducing risk of chronic disease (27). In addition, the Navajo Health and Nutrition Survey (1991–1992), designed to identify priorities and opportunities related to health and nutrition among the Navajo, found that intake of fruit and vegetables was low (less than once per day), whereas intakes of fats and energy from foods such as fry bread, home-fried potatoes, bacon, sausage, and soft drinks were high and provided 41% of total energy (19, 28). Major factors identified as affecting food choice were cost, availability, and shelf life (19, 28).

In addition to dietary risk factors, physical inactivity is prevalent in adults as well. Approximately 51.4% of NA and AN adults aged 18 y or older do not meet federal PA guidelines compared with 44.1% of non-Hispanic whites (29). Other studies have also found low PA and decreased leisure-time activity in NA populations (30). As a result of this unhealthy food environment combined with decreased PA, obesity prevalence has risen to 42.3% of adults aged ≥18 y (31).

There is great potential for large, multilevel, multicomponent (MLMC) interventions to prevent obesity and other diet-related chronic diseases that disproportionately affect NA and AN populations (32, 33). School-based programs (34–41) have resulted in improvements in psychosocial factors such as self-efficacy, intentions, and knowledge. There have also been improvements in dietary intake such as increased fruit and vegetable intake, decreased soft drink consumption (37), and decreased percentage fat intake (38). Food store-based programs (43, 44) have also found positive outcomes, including increased purchasing of healthy foods and improved dietary intake. Those taking more of an MLMC approach (45–47) have found modest success, for example greater improvement in healthy food acquisition scores and health knowledge scores among intervention respondents (45), reduced consumption of discouraged foods and utilization of unhealthy cooking methods (such as pan-frying with added fat) that resulted in a significant increase in the use of healthy food preparation methods over 12 mo (42), and greater reduction in consumption of discouraged high-fat meats, high-fat dairy, refined grains, and unhealthy drinks, as well as decreased overall energy intake in intervention respondents (42). These data provide evidence that nutrition-based interventions in this population are not only feasible but they can be successfully implemented and carried out to yield health benefits. However, despite some positive findings, a sustainable impact on obesity has not been achieved and research has yet to identify an optimal lifestyle intervention to prevent obesity and its related comorbidities in adult NA populations. The studies highlighted here establish the potential for success of community-based, institutional-level interventions, but none combined all ecological levels into 1 study. Taken together, several gaps in the literature can be identified: the need to include worksites and food stores as a part of a multilevel intervention, lack of emphasis on PA, potential use of children as change agents to influence adult behavior change, and the need for community-randomized trials for appropriately testing MLMC interventions.

By working within multiple levels, large MLMC interventions can address several of the factors contributing to obesity burden within these communities, such as low healthy food access and availability, barriers to PA, and low social support for healthy behavioral changes. In addition, given that these tribal communities tend to be small and consisting of few food stores, worksites, and schools, these MLMC interventions can be especially impactful. Entire tribal communities may benefit from such interventions as the potential for individuals to be exposed to the intervention increases dramatically.

The OPREVENT (Obesity Prevention and Evaluation of InterVention Effectiveness in NaTive North Americans; ISRCTN76144389) program was developed to address some of the research gaps, specifically incorporating an MLMC design with the addition of a worksite component and emphasis on PA, and to work towards the development of effective and sustainable obesity prevention strategies for NA adults. The objective of the OPREVENT program was to decrease obesity and diet-related chronic diseases in NA adults through changing the food-purchasing environment, improving nutritional intake, and increasing PA. The aim of this article is to describe the OPREVENT intervention design and provide insight on delivering these types of interventions in rural NA communities.

## Methods

The OPREVENT study implemented a theory-based, culturally appropriate MLMC intervention strategy for its design of an MLMC intervention coordinated across local food stores, schools, worksites, and community media. A community-randomized controlled trial was used to evaluate the impact of the intervention in 5 NA communities. Evaluation instruments were constructed to collect individual, institutional, and process data, and to assess intervention outcomes and impacts.

### Theoretical framework

**FIGURE 1:**
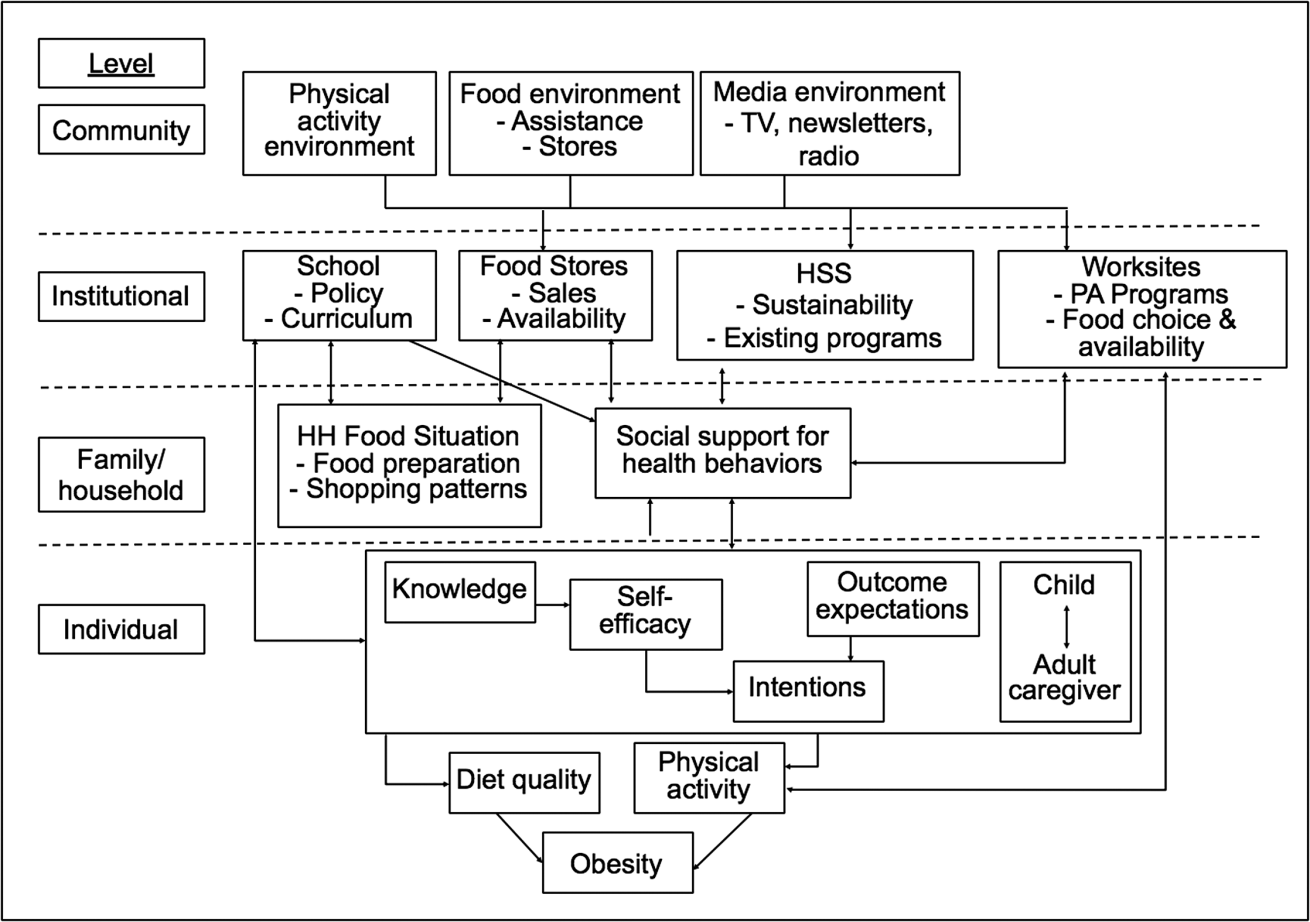
OPREVENT (Obesity Prevention and Evaluation of InterVention Effectiveness in NaTive North Americans) conceptual framework. HH, household; HSS, health and social services; PA, physical activity.

The conceptual framework describing the OPREVENT intervention and evaluation is shown in **Figure 1**. Based on both Social Cognitive Theory (SCT) and the Social Ecological Model (SEM), this framework outlines the constructs and relations at each level of the multilevel design and shows how the intervention components reinforced each other and affected key mediators of diet and PA, as published elsewhere (48). SCT emphasizes triadic reciprocal determinism, or the interaction between people, the behavior, and the environment. It focuses on the individual's ability to change the environment to support a desired behavior as well as the capacity for collective action. The OPREVENT intervention drew on this theory by working within community levels with the capacity for collective action (food stores, worksites, schools, and media) and with behavior change messages targeting the individual. Intervention messaging was also developed with SCT concepts in mind, including changing outcome expectations (likelihood and value of eating healthy foods and exercising), building self-efficacy (supporting people in their ability to perform behaviors and bring about desired outcomes), observational learning (opportunities for community members to observe their peers engaging in healthy behaviors), incentive motivation (rewards for achieving nutrition and PA goals), and facilitation (tools to make new behaviors easier to perform), among others (49). SCT pairs well with SEM, which proposes that individual, interpersonal, community, organizational, and societal factors should each be considered when planning and implementing health promotion interventions (49). The overall approach emphasizes intervening within and across multiple ecological levels to promote sustainable behavior change. This allows for maximum exposure to the intervention, as community members will encounter intervention activities and messages at several levels, helping to reinforce the behaviors being promoted.

The community and institutional levels addressed food and PA resources and local media as part of the broader physical and social environments that affected the 3 institutions targeted by OPREVENT: food stores, worksites, and schools. Food purchasers would be exposed to the food store program, working adults would be exposed to the worksite program, and children would be exposed to the school program and encourage adult members of their households to purchase, prepare, and consume healthier foods and be more physically active. The multiple intervention sites would reinforce each other and build social support for positive lifestyle change within households. These lifestyle changes would occur at the individual level within the household as a “unit” via key psychosocial mediators, leading to changes in diet, PA, and ultimately obesity.

### Setting

To be eligible for the OPREVENT program, tribal communities were required to have an on-reservation population of ≥500, ≥1 on-reservation school, ≥1 on-reservation food store (grocery store, supermarket, or convenience store), and ≥1 worksite with ≥5 tribal member employees. Over 30 letters were sent to administrations of eligible tribal communities within the targeted areas (Upper Midwest and Southwest regions of the United States) introducing the proposed intervention and providing contact information for further information. Ten NA communities expressed interest in participating; 8 were selected to participate in the intervention. This was later reduced to 6 owing to budgetary constraints. We were unable to obtain tribal approvals from 1 of these communities and therefore 5 proceeded with the intervention program. These 5 communities represented 4 different tribal affiliations, with 3 of the communities in the Southwest and 2 in the Upper Midwest. **Table 1** summarizes community demographics of the 5 participating communities, which we will go on to describe in detail. All population statistics were obtained from the US Census website for the year of intervention baseline data collection (50).

**TABLE 1:** OPREVENT (Obesity Prevention and Evaluation of InterVention Effectiveness in NaTive North Americans) community demographics^1^

Community	Population^2,3^	NNA^3^, %	Median household income^3^	Below poverty line^3^, %	High school education^3^, %	Participating food stores, *n*	Participating schools, *n*	Participating worksites, *n*
A	∼400^2^23,548^3^	2.9	$40,373	16.9	90.2	7	1	7
B	∼3700^2^8575^3^	13.9	$39,803	17.3	82.4	5	1	5
C	∼6700^2^39,465^3^	18.5	$36,098	24.2	81.5	5	1	5
D	∼1952^2^17,256^3^	13.7	$34,037	23.5	76.8	4	1	4
E	∼1700^2^27,329^3^	42.1	$34,565	29.2	81.4	4	1	4

^1^NNA, Native North American.

^2^Statistics given for NNA community (48).

^3^Statistics given for county (48).

Community A was the smallest of the 5 communities with an on-reservation population of ∼400 residents at the time of the study. The community was located in the Upper Midwest/Great Lakes region. English was the primary language spoken, although some Elders were able to speak the local tribal language. Community A had a large casino, a convenience store/gas station/take-out pizza restaurant, a health center, a senior center, a cultural heritage center, and a tribal K-12 school. The community was ∼17 miles from a larger town to which many tribal members traveled for grocery shopping and other services. Several community members engaged in traditional activities such as hunting, berry picking, and ceremonies such as Pow-wows.

Community B was also located in the Upper Midwest/Great Lakes region and, at the time of the study, had a population of ∼3700 residents. This community had a large casino, a local radio station, a recreational area for camping and fishing, a senior center, a health center, a library, and a community college, and access to a local nontribal K-12 school. There were 5 food stores serving Community B and a small neighboring town where tribal members could purchase food and other services. Community members were actively engaged in traditional activities including rice harvesting, hunting, berry picking, harvest feasts, and Pow-wows.

Community C was the largest of the 5 communities and located in the Southwest, with ∼6700 residents at the time of the study. There was a large casino, a gas station/convenience store, a tribal school, a wellness center, and a senior center. Because of its proximity to a nearby town with a population of ∼10,000, community members had access to 5 food stores for grocery shopping. English was spoken, but most tribal members also spoke the traditional language. Community members engaged in many traditional activities, such as fishing, ceremonial dances, and their annual feast day.

Community D was located in the Southwest, ∼85 miles southwest of a large urban area (>500,000 residents), and had a population just short of 2000 residents at the time of the study. Community D had a health clinic, a K-12 school, a local radio station, a senior center, a recreational facility, and a gas station/convenience store. Many Elders only spoke the local language, but younger generations spoke English. Community members were actively engaged in traditional activities including sheep herding, Pow-wows, and jewelry making, and traditional foods were consumed frequently.

Community E had ∼1700 residents at the time of the study and was located in the Southwest, 40 miles west of a large urban area (>500,000 residents). There was a health center, a senior center, a tribal school, and an adult learning center. There was 1 small convenience store located on the reservation, but many residents drove the 40 miles to the city for grocery shopping and other services. Most community members spoke the local language as well as English, although there were many Elders who only spoke the local language. Traditional activities such as sheep herding, Pow-wows, and jewelry making were enjoyed by most community members. Traditional foods were commonly consumed.

### Tribal approvals

The tribal approval process began with speaking to tribal community leaders about interest in participating in the study. Letters of support and memoranda of agreement were obtained from schools, school boards, health agencies, and other tribal agencies (e.g., tribal enterprises and worksites) that would be affected by the proposed intervention. Formal presentations were made by team members to the local government authorities where the purpose and proposed activities of the intervention were discussed, after which a formal vote for a resolution was approved. The written proposal was then sent to the tribal or Indian Health Service (IHS) Area Office for approval where it was reviewed and returned to investigators with questions and comments. Once all questions and comments had been addressed and approval obtained, all information, letters of support, and approvals were sent to the tribal or IHS institutional review board (IRB) for approval. Periodic updates were provided for tribal groups and health boards. All research activities were approved by the IHS IRB, the Navajo Nation Human Research Review Board, and the Johns Hopkins IRB.

### Formative phase

OPREVENT was aimed at designing and implementing a community-driven approach to intervention design. To accomplish this, trained team members conducted a comprehensive formative assessment using a variety of qualitative approaches between the summer of 2010 and the summer of 2011 across 4 of the 5 communities (1 of the 5 participating communities chose not to participate in the formative evaluation). The aim of the formative assessment was to understand the local contexts of obesity in the study communities, to identify biocultural, political-economic, and environmental factors either contributing to or combatting the obesity problem, and to work with community members to identify problem foods and behaviors in each community and develop key intervention messages and materials. This process allowed community members to participate and develop a sense of ownership of the intervention program as well as to ensure that all materials and strategies were culturally appropriate and acceptable (51–53).

With a team of trained graduate students and locally hired NA staff, we engaged in purposive and snowball sampling to recruit participants across worksites, health clinics, schools, and tribal agencies. Using purposive sampling, all adults 18 y and older with ≥1 child living in their household 6 y or older, and their child or children were eligible to participate. We drove and walked to appropriate sites to invite community members at large, as well as institutional administrators, managers, and staff across sectors. Team members identified local worksites and schools, the health clinic, the tribal council, the local casino, and gas stations nearby and by driving or walking on foot, we introduced ourselves to community stakeholders and invited them to participate. For more information on the details of our formative assessment methodology, please refer to previously published articles by Gadhoke et al. (51), Gadhoke et al. (52), and Christiansen et al. (53).

Briefly, a total of 168 adults and children in the Upper Midwest and 82 adults and children in the Southwest participated in the formative assessment (52). Formative techniques included participant observation (through journaling and notetaking as attendees of local ceremonies, events, feasts, and other celebrations by PG and KC), as well as in-depth interviews, focus groups, and community workshops.

Adult in-depth interviews took place at the respondents’ homes, tribal health clinics, worksites, schools, and community centers. Focus groups of 8–13 adults were administered at schools (both public and Native schools), casinos, and community centers. In addition, some household group interviews were conducted to learn more about the intergenerational household as it may have been specifically related to the intervention's school component. These interviews were administered by 1 coauthor (PG) upon invitation only by key respondents in 1 of the Upper Midwest communities. Community workshops were administered at the initiation and at the conclusion of the formative assessment to member-check findings and provide feedback on intervention materials across the 4 institutional levels. In addition, we conducted paired-child interviews at tribal health centers (with children pairs and child–adult pairs) specifically for the school component. For more information on the formative assessments, please refer to Gadhoke et al. (52) and Christiansen et al. (53).

**FIGURE 2:**
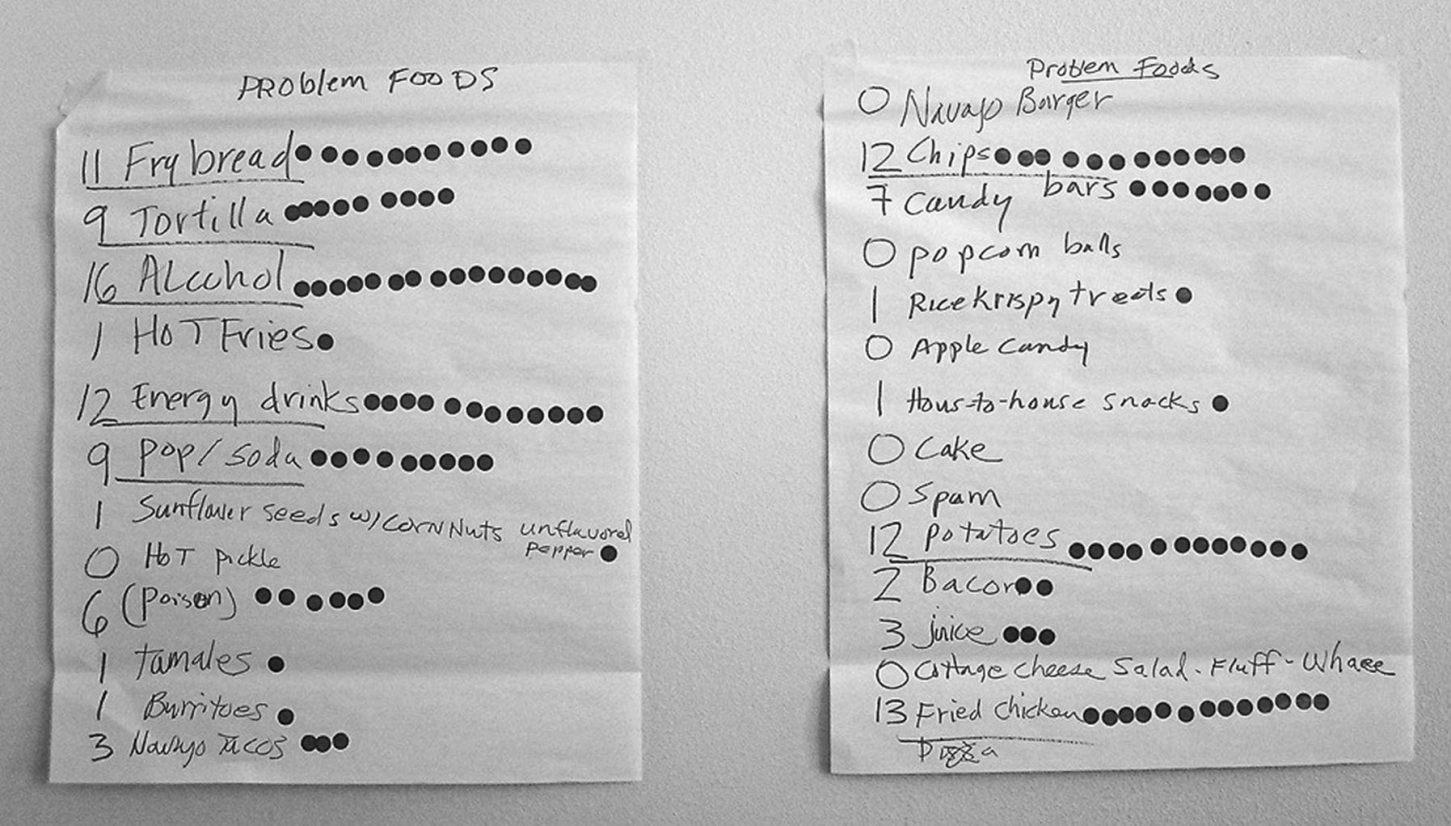
Problem foods identified during a community workshop in community D.

During community workshops, key community health issues and concerns were discussed and participants *1*) identified salient health issues for the community; *2*) prioritized problem foods; *3*) prioritized acceptable alternatives to these foods; *4*) prioritized unhealthy food behaviors; *5*) prioritized alternative behaviors to promote; *6*) identified preferred modes of communication (newsletters, radio, presentations, billboards, meetings, etc.); and *7*) assisted in the development of culturally appropriate health messages for the community. For example, to identify problem foods, workshop participants were asked to list all problem foods and beverages in the community. Once a list was formed, each participant was asked to put a sticker next to the foods and beverages that they felt were truly of highest priority. Results of this exercise from Community D are shown in **Figure 2**. This process was repeated in each community and a final list of problem foods and beverages was used to inform intervention messages and activities. Feedback was also used to ensure culturally appropriate content, for example promotion of traditional forms of PA like hunting and canoeing, use of storytelling to communicate lessons in the school curriculum, and promotion of traditional foods like wild rice and game meat.

Key findings from the formative phase have been published elsewhere. These include a summary of children acting as change agents for adult food and PA in NA households in the Upper Midwest (51) and a qualitative study on women's coping strategies for obesity risk–reducing behaviors in NA households (52, 53). Findings from these analyses helped to inform the structure of the school component to focus on children as change agents, as well as to guide the development of specific intervention messages such as working together with friends and family to improve household food and PA habits.

Findings from community workshops and the formative phase were also utilized in the development and modification of the study impact evaluation instruments, which were initially based on surveys implemented in previous work in NA and AN communities (32, 54, 55) and then tailored to the specific communities of OPREVENT. Community experts provided feedback on survey content and wording. Pretesting and piloting in both regions were completed to further refine the impact evaluation tools. Evaluation instruments are described in detail in the “Measurements” section.

Feedback was also solicited for intervention materials design. Local and/or independent graphic artists were commissioned to design all graphics for the OPREVENT materials, which were further modified based on community feedback. The school curriculum was developed by a team of NA curriculum developers and the research team through regular school working group remote meetings. Characters appearing throughout the school curriculum were given names common to the participating tribes, based on community input, and drawn to be representative of the communities.

Compensation for participation in the formative phase was provided in the form of gift cards. Adults were compensated with $20 gift cards and child-paired interviews were compensated with $10 gift cards. Adult community workshop participants also received a $20 gift card for their time and contributions.

### The intervention

**Table 2** summarizes the intervention in more detail, including phases, duration, key messages, and the foods and behaviors promoted within each phase.

**TABLE 2:** OPREVENT (Obesity Prevention and Evaluation of InterVention Effectiveness in NaTive North Americans) intervention by phase^1^

Phase	Duration	Key messages and behaviors	Promoted foods and beverages	Activities by component
1. Choose Wisely	4 wk	Lower in sugar (<10 g sugar per serving); think before you drink; visit worksite water station makeovers	- Water - Diet soda - Reduced-sugar drink mixes - 100% juice - Indian tea	- Food store: “Lower Sugar” shelf label; nonsugar drink mixes, diet sodas, and flavored waters taste test; low-sugar beverage educational display; posters, flyers, and booklets; giveaways: calendar, button, shopping list notepad, reusable shopping bag, and water bottle - Worksite: water station stocked with water filter, sugar-free drink mixes, and water bottles; posters, flyers, and booklets - School: none - Media: radio announcements; newsletter
2. Make a Plan, Set a Goal	4 wk	Lower in fat (<10% daily value of fat per serving); bring a healthy lunch to work; plan ahead for shopping and meal prep; lower in fat meal prep; use nutrition labels when shopping	- Cooking spray - Low-fat bologna or turkey luncheon meat - 100% whole-wheat bread - Fresh fruit - Low-fat or fat-free mayonnaise	- Food store: “Lower in Fat” shelf label; pancakes with cooking spray, and healthy sandwiches taste test; “Shop Healthy” educational display; posters, flyers, booklets, and recipe cards; giveaways: buttons, potholders, and lunch bags - Worksite: maintain water stations; stock coffee stations with zero-calorie sweeteners, low-fat creamers, and powdered milks; posters, flyers, and booklets - School: piloted June 2012 - Media: radio announcements; newsletter
3. One Step at a Time	4 wk	Higher in fiber (>10% daily value of fiber per serving); let's get active; exercise with a buddy; increase daily steps	- Fresh fruit - Canned fruit in light syrup or 100% fruit juice - Water	- Food store: “Higher in Fiber” shelf label; fresh fruit or fruit canned in light syrup or 100% fruit juice taste test; “Let's Get Active” educational display; posters, flyers, booklets, and recipe cards; giveaways: buttons, PA diaries, Frisbees, and pedometers - Worksite: pedometer challenge kick-off; interactive lunch sessions; maintain water and coffee stations; posters, flyers, and booklets - School: teacher training sessions November–December, 2012 - Media: radio announcements; newsletter
4. Make it Count, Make it Last	4 wk	Lower in sodium (<10% daily value of sodium per serving); track your food and PA; be aware of portion sizes; rinse canned vegetables	- Low-sodium pretzels and crackers - Low-sodium nuts - Low-sodium canned vegetables - Dried beans	- Food store: “Lower in Sodium” shelf label; low-sodium pretzels and crackers taste test; rinsing canned vegetables demo; “Make it Count, Make it Last” educational display; posters, flyers, booklets, and recipe cards; giveaways: calendars and PA diaries - Worksite: pedometer challenge; interactive lunch sessions; maintain water and coffee stations - School: curriculum used for instruction - Media: radio announcements; newsletter
5. Live Life in a Good Way/Celebrate the New You	4 wk	Healthier choice (healthier snack alternatives); share your success; take care of your body, mind, and spirit; sustain behavior change; choose healthier snacks; track your food	- Granola bars - Sugar-free/low-fat Jell-O pudding - Baked chips - Graham crackers	- Food store: “Healthier Choice” shelf label; granola bars, baked chips, sugar-free and low-fat Jell-O, rice cakes, and Graham crackers taste test; “Live Life in a Good Way” educational display; posters, flyers, booklets, and recipe cards; giveaways: food diaries - Worksite: working with managers to continue and sustain pedometer challenge, water stations, and coffee stations; posters, flyers, and booklets - School: curriculum used for instruction - Media: radio announcements; newsletter

^1^PA, physical activity.

The intervention was implemented in 5 phases: Phase 1—Choose Wisely; Phase 2—Make a Plan, Set a Goal; Phase 3—One Step at a Time; Phase 4—Make it Count, Make it Last; and Phase 5—Live Life in a Good Way/Celebrate the New You. Each phase focused on specific target foods (as identified by the communities via formative research) and associated food-related behaviors such as cooking or meal planning. PA was also a focus of each phase, although with lower intensity.

Based on formative research, acceptable alternatives to problem foods were promoted within the communities. These included a combination of fruits, vegetables, whole-grain products, low-fat snacks, and low-calorie beverages. Key promoted behaviors included portion control, shopping on a budget, and other related household coping strategies. Promotional materials such as posters and flyers promoted these foods, whereas educational displays and booklets provided more detailed information and education. Interventionists also conducted taste tests and cooking demonstrations to promote these foods. Promoted foods were identified with shelf labels to make them easier to identify for consumers.

Another key behavior change was increasing PA. Promotional materials provided educational information on types and intensities of PA and suggestions for success such as setting goals and tracking progress, exercising with friends, and getting the whole family involved. Flyers were also used to promote the worksite pedometer challenges.

Interventionists were local tribal community members when possible. Interventionist trainings were held at the beginning of each phase to provide information on new materials, promoted foods, and activities specific to each phase. Weekly team meetings were held to assess intervention progress and questions related to intervention delivery.

**FIGURE 3:**
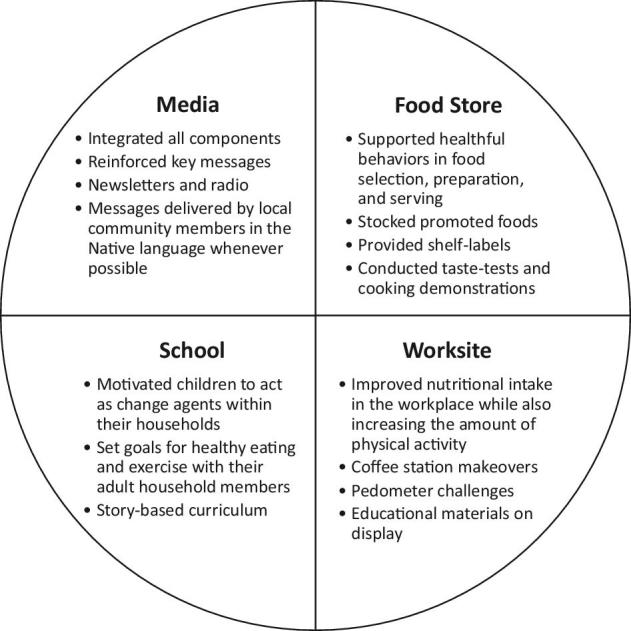
OPREVENT (Obesity Prevention and Evaluation of InterVention Effectiveness in NaTive North Americans) intervention components.

The primary activities of each OPREVENT intervention component are summarized in **Figure 3**. Food stores, worksites, and schools were recruited from each community and, in return for volunteering to participate, received intervention materials and support from the Johns Hopkins Bloomberg School of Public Health (JHSPH) study team. All community members had the potential to be exposed to the intervention.

The objective of the OPREVENT food store program was to create a food-purchasing environment that supported the health and nutritional well-being of community members. Intervention stores were asked to stock and promote certain healthy foods. In turn, interventionists would promote these foods by conducting taste tests and cooking demos in store; these foods were promoted through intervention materials in-store including posters and shelf labels. Promoted foods were chosen based on healthy foods that were already acceptable and available in the intervention food stores but were of equal or lesser cost than their less healthy alternatives (43). All owners, managers, and staff of the intervention food stores attended trainings to implement the changes, which emphasized keeping all signage visible, placing promoted foods in easily accessible locations, and how to answer questions related to OPREVENT activities.

The OPREVENT worksite program objective was to improve the food environment and nutritional intake in the workplace while also increasing employees’ PA. Local worksites such as food stores, government offices, and casinos were recruited for this component. Coffee station makeovers were implemented throughout the intervention, for which less healthy options, such as whole milk and sugar, were replaced with healthier alternatives, such as skim milk and zero-calorie sweeteners. Worksites were also encouraged to offer healthier selections in vending machines and adopt policies to ban sugar-sweetened beverages from being sold on-site. PA was encouraged via the implementation of pedometer challenges. Pedometers were distributed to employees who were interested in participating and weekly monitoring of steps was encouraged. Prizes were awarded to individuals who met pre-established goals (51).

The OPREVENT school program was developed to integrate with the food store, worksite, and media components. Parental permission and child assent were obtained for participation. The school program was implemented in the second to sixth grades of participating schools and composed of 4 grade-specific curricula and storybooks (1 each for grades 2–4 and 1 for grades 5–6). Each curriculum was composed of 16 weekly, 45-min teacher-led units/lesson plans that began with a story excerpt to introduce the main intervention teaching concepts and continued with hands-on learning activities and in-class PA breaks. The storybooks followed the story of an extended NA and AN family as the father is diagnosed with type 2 diabetes. In the storybooks, the family learns about nutrition, PA, and prevention of diabetes risk factors such as obesity. Children in the story act as change agents to influence positive health behaviors relating to food and PA in adults. The fifth- and sixth-grade components also included NutriBee, a nutrition intervention incorporating Bee-style games into a 20-h school program of 10 modules, and focused more on student-initiated activities such as media awareness (56).

The school curriculum was designed to educate children in the classroom and motivate them to act as change agents within their households. The child-as-change-agent approach to adult obesity prevention involves a systematic, evidence-based framework that does the following: *1*) provides children and youth with knowledge on health and nutrition from a holistic perspective that is culturally sensitive for the engaged NA communities; *2*) builds children’s and youths’ self-efficacy for knowledge sharing with their adult caregivers; *3*) structures continuous, ongoing knowledge-building and sharing with children and youth and their adult caregivers weekly; *4*) builds social support in the household (with the child or children as social support for adult caregivers) through change agency for adult nutrition and PA; *5*) process evaluation to measure the extent of dose, frequency, and fidelity of the program within the classroom at the individual child or youth level; and *6*) impact assessment of adult knowledge, self-efficacy, psychosocial factors, food-related and PA-related behaviors, and BMI (pre- and postintervention, which is the school-based curriculum). A focused, systematic, and methodical evidence-based approach was taken to train children to be empowered and motivated to act as change agents in their households for adult obesity prevention through nutrition and PA. Students’ participation was evaluated through daily classroom process evaluation as well as students’ impact as measured through pre-post adult impact survey questionnaires and anthropometry. Children were encouraged to share what they learned from the curriculum with their family members, request healthy foods for meals and snacks, and encourage family PA and togetherness. To encourage children as change agents, strategies such as family-oriented community kitchen activities, informational booths during parent–teacher nights, and newsletters were employed. Schools were also encouraged to consider policy changes such as banning sugar-sweetened beverages and fatty snacks (51).

The OPREVENT media program consisted primarily of radio announcements and newsletters, and was designed to integrate with the school, food store, and worksite components while reinforcing the key intervention messages, which appeared in posters, brochures, flyers, and/or booklets throughout each of these community sites. These messages varied by phase (see Table 2) and included using food labels to help identify healthier choices in community stores, taking advantage of work breaks and free time to be physically active with friends, and choosing water instead of sugar-sweetened beverages. Upcoming community events such as health fairs, community walks, and the food store taste tests and cooking demonstrations were also announced. Newsletters were mailed to intervention community members in the Upper Midwest communities and radio announcements were broadcast in intervention communities in the Southwest communities. Messages were delivered by local community members and in the Native language whenever possible.

### Individual impact evaluation

To evaluate the intervention, a community-randomized controlled trial was conducted in the 5 participating NA tribal communities. The 5 communities were randomly assigned to either Immediate (*n* = 3) or Delayed (*n* = 2) Intervention; Immediate Intervention communities received the OPREVENT program beginning in early summer of 2012, immediately after the conclusion of baseline data collection. Delayed Intervention communities received the OPREVENT program beginning in the fall of 2015, after the conclusion of follow-up data collection.

Individuals were recruited for the individual-level evaluation from each participating community. Eligible individuals were required to *1*) have lived in their current household for at least the last 30 d; *2*) be NA adults aged 18–65 y; *3*) be tribal members; and *4*) be the main food shopper or preparer of their household. This final criterion was included because several of the intervention messages were targeted to behavior change at the point of purchase, and formative work identified the main food shopper or preparer as the individual with the most influence over dietary intake within the household. Exclusion criteria included pregnant women at the time of the study. Households within each community were randomly selected from tribal lists, and 1 eligible adult was randomly selected from each household. If the eligible adult declined to participate, recruitment continued with the next household on the list until the target enrollment was achieved. Signed consent was obtained from all respondents, and compensation was provided in the form of $40 Walmart gift cards after the baseline and follow-up interviews.

Sample size was calculated based on the original design for 8 communities, a power of 80%, and type I error of 5%. Pilot data from 10 NA and First Nations communities were used to calculate intraclass correlation (ICC) and within-community variance for primary outcomes of interest: energy (in calories), servings of fruits and vegetables, percentage calories from fat, and percentage time on sedentary activity. The ICC was negative for energy, vegetable servings, and percentage calories from fat; however, a conservative approach was utilized, and the detectable difference was calculated based on a range of ICCs from 0.001 to 0.01 and sample sizes from 40 to 70 per community. The ICC for fruit servings and percentage time on sedentary activity was 0.01 and 0.31, respectively. A final sample of 50 respondents per community would allow detection of: a decrease of ∼320 calories, a change in 4% calories from fat, 14% change in percentage time on sedentary activity, and at least a 1-serving increase in the number of fruits or vegetable servings in the Immediate Intervention group compared with the Delayed Intervention group. To account for loss-to-follow-up and nonparticipation (∼20% from previous work), 60 respondents/community were recruited. This was later increased to 85 respondents/community as the total number of communities was reduced from 8 to 5 before intervention implementation. Impact analysis has also been adjusted to accommodate for this potential limitation, and more conservative approaches are being used (analysis is in progress).

It was hypothesized that, by the end of the 1-y intervention, respondents in the 3 Immediate Intervention communities would have *1*) improved dietary intake (as measured by energy intake, percentage fat intake, increased fruit and vegetable consumption, and increased fiber intake); *2*) improved PA habits (increased days per week engaged in PA, increased time per week engaged in PA, increased PA levels, and decreased time spent sitting); and *3*) improved psychosocial factors and food- and PA-related behaviors including self-efficacy, knowledge, and intentions as compared with respondents in the Delayed Intervention communities.

### Measurements

The OPREVENT intervention was designed to be evaluated at the individual and institutional levels. Further process evaluation was designed to assess the extent of implementation dose, reach, and fidelity. Data collection instruments are summarized in **Table 3**.

**TABLE 3:** OPREVENT data collection instruments^1^

Data type	Data collection instrument	Collection	Outcome variables
Impact	Adult Individual Impact Questionnaire	Pre-post intervention	Knowledge; self-efficacy; intentions; outcome expectations; food purchasing frequency; food preparation; social support; medical history; demographics
			Anthropometry: height; weight; BMI; waist and hip circumference; percentage body fat
			PA: days per week, time per week, and MET-minutes per week spent in sitting, moderate-intensity PA, and vigorous-intensity PA; PA level (low, moderate, high)
	Semiquantitative food-frequency questionnaire	Pre-post intervention	Cereals, dairy, rice/pasta, vegetables, fruits, meals, desserts and snacks, beverages, alcohol
	Food Store Impact Questionnaire	Pre-post intervention	Stocking and sales; outcome expectations
	Worksite Impact Questionnaire	Pre-post intervention	PA policies and resources
	School Impact Questionnaire	Pre-post intervention	Food and PA policies; self-efficacy; intentions
Process	Food Store Environmental Checklist	Once per phase	Presence of OPREVENT-promoted foods
	Food Store Process Form	Once per phase	Presence of OPREVENT promotional materials including posters and shelf labels
	Worksite Environmental Checklist	Once per phase	Availability of healthy foods and resources
	Teacher Checklists	Once per week	Lesson completion; general feedback
	Mass Media Process Form	Once per phase	Presence of promotional materials; number of radio announcements and newsletters delivered per phase
	Interventionist Site Visit Form	One per visit	Reason for visit; interactions; activities completed
Exposure	Intervention Exposure Evaluation	Postintervention	Exposure to intervention materials, activities, and components

^1^MET, metabolic equivalent; OPREVENT, Obesity Prevention and Evaluation of InterVention Effectiveness in NaTive North Americans; PA, physical activity.

#### Individual-level data collection

The Dietary Assessment Questionnaire included a brief semiquantitative food-frequency questionnaire (SFFQ) and a 24-h recall and was implemented at baseline and follow-up. The SFFQ was adapted from SFFQs developed from 24-h dietary recalls in Canadian First Nations in Northwestern Ontario and the Navajo Nation (32, 54, 55). The questionnaire was brief, at only 45 items, and covered the last 30-d period. Foods promoted and discouraged by the OPREVENT program were included on the SFFQ, and frequency of consumption was reported using 8 different categories, ranging from “Never” to “Two or three times a day” (32, 55). Amounts consumed were reported using familiar household units (such as bowls and spoons) or food models representing locally available portion sizes (32, 55). The 24-h recall was administered using the 5-step multiple-pass approach.

The Adult Impact Questionnaire (AIQ) was a 140-question evaluation instrument used to assess individual behavior and potential mediators and moderators of diet and PA at baseline and follow-up. Although the AIQ was not validated in the target population, its development was directly informed by the extensive formative phase. Scales included *1*) knowledge: respondent's knowledge regarding health behaviors emphasized by OPREVENT; *2*) self-efficacy: respondent's confidence to perform various healthy behaviors; *3*) intentions: respondent's intentions to perform various healthy behaviors; and *4*) outcome expectations: respondent's perceived benefits of healthy diet and PA. In addition, the AIQ assessed multiple household-level outcomes, including *1*) food purchasing frequency: healthy food purchasing score based on purchases of OPREVENT-promoted foods in the last 30 d; *2*) food preparation: food preparation methods for commonly consumed foods in the last 30 d and overall healthiness of food preparation score; and *3*) social support: 4 dimensions of family and social support for healthy food and PA behaviors.

The AIQ included PA estimates using the modified short-format International Physical Activity Questionnaire (IPAQ-SF). The IPAQ-SF was used to assess days per week, time per week, and metabolic equivalents (MET)-minutes per week engaged in all levels of PA as well as PA level (low, moderate, high). The IPAQ-SF assesses 3 intensities of activity—walking, moderate intensity, and vigorous intensity—and separate scores are provided for each (57). Based on formative research, the descriptions and examples of moderate and vigorous intensity activity in the IPAQ-SF were modified to be more culturally relevant (e.g., shoveling snow was added as a culturally relevant example of “vigorous” PA). An additional question asking whether the level of activity reported in the last 7 d was less than average, average, or more than average was also added.

Sociodemographic variables collected via the AIQ included age, sex, household size, marital status, educational level, employment status, current smoking status, personal and family history of chronic disease, and food-assistance program participation.

For anthropometry, body weight was measured twice using a Tanita 300GS (Tanita Corp.) to the nearest 0.1 pound (and a third time if different by >5.0 pounds). Height was measured twice to the nearest 0.5 inch using a stadiometer (and a third time if different by >0.5 inches). Body composition was measured twice via bioelectrical impedance analysis using a Tanita 300GS (Tanita Corp.). Waist circumference was measured twice using a retractable measuring tape to the nearest 1 cm (0.4 in) [and a third time if different by >5 cm (2.0 in)]. Hip circumference was measured twice using a retractable measuring tape to the nearest 1 cm (0.4 in) [and a third time if different by >2 cm (0.9 in)]. Measurements were done twice to allow for averaging of the final accepted value.

The Intervention Exposure Evaluation (IEE) was used to assess participant exposure to the intervention components. The IEE was administered once at follow-up to all participants in both Immediate and Delayed Intervention communities. The questions were designed to measure variation in exposure based on participant use of community media, shopping frequency at participating food stores, number of children in the second to sixth grades, employment status, and participation in community events and activities. Respondents were shown intervention materials from each component of the intervention and asked whether they recognized and/or acted upon the materials. Red-herring questions were included to assess the validity of respondents’ answers. Exposure scores were developed using these data.

#### Institutional-level data collection

Impact questionnaires were also used to assess each institutional-level intervention component: Food Store Impact Questionnaire, Worksite Impact Questionnaire, and School Impact Questionnaire. The Food Store Impact Questionnaire assessed typical stocking and sales of OPREVENT-promoted foods, outcome expectations of healthier food item sales, and outcome expectations of promotional activities’ impact on sales (e.g., shelf labels, cooking demos, and taste tests). The Worksite Impact Questionnaire assessed policies to support health and PA behavior within the worksite and available resources to encourage PA. The School Impact Questionnaire assessed policies to support healthy food and PA within schools, self-efficacy for incorporating health education into the curricula, and intentions to incorporate health education into the curricula.

#### Process evaluation

Process data were measured for the duration of the intervention, at least once per phase. Data collectors and interventionists collected these data for each of the intervention components and activities. Food store–level data included the Food Store Environmental Checklist and the Food Store Process Form. The former was used to track whether the OPREVENT-promoted foods were in stock at all participating food stores, and the latter was used to track the presence of OPREVENT promotional materials including posters and shelf labels. Worksite-level data included the Worksite Environmental Checklist, which was used to track available services at participating worksites (e.g., vending machines, cafés, PA facilities), the availability of OPREVENT-promoted foods in vending machines and other food retailers at worksites, and the presence and quality of items that could help or hinder OPREVENT behavior change messages (e.g., walking paths, break rooms with TVs and DVD players that could be used for workout videos, coffee stations stocked with OPREVENT-promoted items such as calorie-free sweeteners, water stations). Media-level data included the Mass Media Process Form to track the presence of promotional materials throughout each community and record the number of radio announcements and newsletters delivered for each phase. School-level process data were collected using Teacher's Curriculum Checklists for each grade. Teachers reported whether each lesson had been taught and provided general feedback and comments. Finally, process data were also collected for each site visit and interactive session using the Interventionist Site Visit Form. Interventionists recorded the reason for each visit, the people with whom they met, and the activities completed. If the visit included an interactive session, they also recorded the number of consumers contacted (reach); the number of items given away, such as recipe cards, taste test samples, or promotional giveaways (dose); and how well each session was delivered (fidelity).

## Discussion

The OPREVENT intervention was an innovative and partnered approach to the reduction of obesity and other diet-related diseases. It was implemented in Immediate Intervention communities from April, 2012 to May, 2013. There was a minor delay in postintervention data collection owing to budgetary constraints; however, all data collection was completed by May, 2014 with an overall retention rate of 70%, for a final *n* = 299. Individual, institutional, and process data are being analyzed to determine intervention impact and overall exposure as well as dose, reach, and fidelity. The MLMC design in combination with multiple levels of data will allow us to determine which intervention components were the most impactful, the behavior change strategies that were most successful, and where there is need for improvement. The study's comprehensive formative research component and community-centered, iterative approach towards evaluation instrument development helped promote high-quality data collection. It also provided a locally contextualized foundation for development of a culturally appropriate program based on the current health attitudes and perceptions within each NA community, and stakeholder involvement supported successful delivery. Involvement of multiple components at several environmental levels resulted in a strong intervention design that served to influence dietary intake, PA, and related factors in a cohesive and reinforcing way.

To our knowledge, only 1 other intervention had utilized such a comprehensive and culturally relevant and sensitive MLMC approach close to that employed in OPREVENT at the time of implementation (47). However, this is the first intervention of its kind to address the problem on a large scale and across 5 communities representing vastly different regions and cultures. The diversity of communities was an added challenge, but one that was addressed through the extensive formative phase to guarantee that intervention messages and materials resonated across all OPREVENT communities.

Results from the evaluation of this program will provide evidence on the effectiveness of large, MLMC adult obesity interventions in NA populations. In addition, the MLMC design contributes to the critical development of evidence-based practices to address health disparities for NAs by addressing their social determinants of health on multiple levels of influence (56). The methods described here directly address the NIH priority of dissemination and implementation science, with the intent to spread information and the associated evidence-based interventions (58). Documentation and dissemination of MLMC research methods in these populations promote the integration of research findings into policy and practice.

Preliminary results of OPREVENT have already been utilized to inform the development of OPREVENT2 (NCT02803853). This follow-up study will take place within 6 new NA communities in the Southwest and Upper Midwest United States. Changes in study design include updated intervention content based on the latest evidence-based practices for nutrition and PA, greater emphasis on PA across all phases and components, greater incorporation of SCT and SEM into intervention messages, the addition of both social media and community action (policy) components, and direct targeting of the evaluation sample through recruitment of worksites where surveyed participants are known to work as well as home mailings for each phase. More rigorous implementation standards have been set to ensure greater exposure to the intervention materials. The OPREVENT2 study will also recruit a larger evaluation sample at 100 adults/community for a total *n* = 600. It is our vision that implementing these changes and modifications based on information acquired from OPREVENT will make OPREVENT2 stronger, successful, and more sustainable, leading to lasting improvements in diet and PA, and overall reduction in health disparities, particularly obesity, in NA adults and households.

## Notes

Supported by USDA National Institute of Food and Agriculture grant #2010-85215-20666 (to JG).

Author disclosures: LCR, BJ, PG, DTC, KC, MP, JS, HP, LEC, and JG, no conflicts of interest.

Published in a supplement to *Current Developments in Nutrition*. This article appears as part of the supplement “Proceedings of the First and Second Annual Conferences on Native American Nutrition,” sponsored by the Shakopee Mdewakanton Sioux Community's Seeds of Native Health campaign through a gift to the University of Minnesota. The guest editors of the supplement, Treena Delormier and Mindy Kurzer, have no conflicts of interest. The opinions expressed in this publication are those of the authors and are not attributable to the sponsors or the publisher, Editor, or Editorial Board of *Current Developments in Nutrition*.

Abbreviations used: AIQ, Adult Impact Questionnaire; AN, Alaska Native; FDPIR, Food Distribution Program on Indian Reservations; ICC, intraclass correlation; IHS, Indian Health Service; IPAQ-SF, International Physical Activity Questionnaire-Short Form; IRB, institutional review board; MLMC, multilevel, multicomponent; NA, Native American; OPREVENT, Obesity Prevention and Evaluation of InterVention Effectiveness in NaTive North Americans; PA, physical activity; SCT, Social Cognitive Theory; SEM, Social Ecological Model; SFFQ, semiquantitative food-frequency questionnaire.
